# On a Monotone Scheme for Nonconvex Nonsmooth Optimization with Applications to Fracture Mechanics

**DOI:** 10.1007/s10957-019-01545-4

**Published:** 2019-07-08

**Authors:** Daria Ghilli, Karl Kunisch

**Affiliations:** 1 Institute for Mathematics and Scientific Computing, Karl-Franzens University, Graz, Austria; 2 Johann Radon Institute, Linz, Austria

**Keywords:** Nonsmooth nonconvex optimization, Monotone algorithm, Fracture mechanics, Sparse recovery, 49M05, 49Kxx, 65Kxx, 74Rxx, 74Pxx, 49J53, 49K99

## Abstract

A general class of nonconvex optimization problems is considered, where the penalty is the composition of a linear operator with a nonsmooth nonconvex mapping, which is concave on the positive real line. The necessary optimality condition of a regularized version of the original problem is solved by means of a monotonically convergent scheme. Such problems arise in continuum mechanics, as for instance cohesive fractures, where singular behaviour is usually modelled by nonsmooth nonconvex energies. The proposed algorithm is successfully tested for fracture mechanics problems. Its performance is also compared to two alternative algorithms for nonsmooth nonconvex optimization arising in optimal control and mathematical imaging.

## Introduction

In this paper, we investigate a class of nonconvex and nonsmooth optimization problems, where the penalty is the composition of a nonsmooth nonconvex mapping with a linear operator and the smooth part is a least square-type term.

Similar optimization problems, in the case where the operator inside the penalty coincides with the identity matrix, have attracted increasingly attention due to their applications to sparsity of solutions, feature selection, and many other related fields as, e.g. compressed sensing, signal processing, and machine learning (see, e.g. [1, 2]). The convex nonsmooth case of the $\ell ^1$ norm has gained large popularity and has been thoroughly studied. The convexity allows to formulate efficient and globally convergent algorithms to find a numerical solution. Here, we mention [3, 4] where the basis pursuit and the Lasso problems were introduced to solve $\ell ^1$ minimization problems.

Recently, increased interest has arisen towards nonconvex and nonsmooth penalties, such as the $\ell ^\tau$ quasi-norm, with $\tau$ larger or equal to zero and less than 1 (see, e.g. [5–10]), the smoothly clipped absolute deviation (SCAD) [11, 12], and the minimax concave penalty (MCP) [12, 13]. The nonconvexity has been shown to provide some advantages with respect to the convex models. For example, it allows to require less data in order to recover exactly the solution (see, e.g. [14–16]) and it tends to produce unbiased estimates for large coefficients [11, 17, 18]. Note that all the previously mentioned works deal with the particular case where the operator coincides with the identity.

Nonconvex optimization problems as we consider, where the operator inside the penalty is different form the identity, arise also in the modelling of cohesive fractures in continuum mechanics, where the concavity of the penalty is crucial to model the evolution of the fracture energy released within the growth of the crack opening. Here, the operator is of importance to model the jump of the displacement between the two lips of the fractures. We refer to [19–22] and Sect. 3.1 for more details.

The study of these problems for nonconvex penalties, including $\ell ^\tau$, with $\tau$ strictly positive and less than 1, the SCAD and the MCP functionals, and for linear operators not necessarily coinciding with the identity, is also motivated by applications different from those arising in fracture mechanics. For example, in imaging the $\ell ^\tau$ quasi-norm, with $\tau$ strictly positive and less than 1, of the numerical gradient of the solution has been proposed as a nonconvex extension of the total variation (like TV) regularizer (see, e.g. [6, 10]) in order to reconstruct piecewise smooth solutions. The SCAD and the MCP penalties have been used for high-dimensional regression and variable selection methods in high-throughput biomedical studies [23]. We mention also that the SCAD has been proposed as a nonconvex penalty in the network estimation to attenuate the bias problem [24].

The main difficulties in the analysis of these problems come from the interplay between the nonsmoothness, the nonconvexity, and the coupling between coordinates which is described by the operator inside the penalty. Since standard algorithms are not readily available, the resolution of these problems requires the development of new analytical and numerical techniques.

In the present paper, we propose a monotonically convergent algorithm to solve this kind of problems. This is an iterative procedure which solves the necessary optimality condition of a regularized version of the original problem. A remarkable property of our scheme is the strict monotonicity of the functional along the sequence of iterates. The convergence of the iteration procedure is proved under the same assumptions that guarantee the existence of solutions.

The performance of the scheme is successfully tested to simulate the evolution of cohesive fractures for several different test configurations. Then, we turn to an issue of high relevance, namely the comparison between two alternative algorithms, the GIST “General Iterative Shrinkage and Thresholding” algorithm for $\ell ^\tau$ minimization, with $\tau$ strictly positive and less than 1, and the FISTA “Fast Iterative Shrinkage-Thresholding Algorithm” for $\ell ^1$ minimization. The comparison is carried out with respect to the infimal value reached by the iteration procedure and with respect to computing time. Our results show that the monotone algorithm is able to reach a smaller value of the objective functional that we consider when compared to the one of GIST. Note that differently from GIST, the monotone scheme solves a system of nonlinear equations at each iteration level. We remark that in [25], GIST was compared with the IRLS “iterative reweighted least squares” algorithm, which is another popular scheme for $\ell ^\tau$ minimization, with $\tau$ strictly positive and less than 1. The results of [25] show that GIST and IRSL have nearly the same performance, with only one difference which is speed, where GIST appears to be the faster one.

An analogous procedure to the one proposed in the present paper was developed in [20] to solve similar problems where the nonconvex penalty coincides with $\ell ^\tau$ quasi-norm, with $\tau$ strictly positive and less than or equal to 1. With respect to [20], in the present paper, we deal with more general concave penalties. Moreover, we carry out several numerical experiments for diverse situations in cohesive fracture mechanics, comparing the behaviours for different concave penalties such as the SCAD, the MCP, and the $\ell ^\tau$ penalty, with $\tau$ strictly positive and less than 1. Finally, in the present paper, we compare the performance of the scheme with that of GIST.

Let us recall some further literature concerning nonconvex nonsmooth optimization of the type investigated in the present paper. In [12, 26], a primal-dual active set-type algorithm has been developed; in the case, the operator inside the penalty coincides with the identity. For more references, in this case, we refer to [20]. Concerning $\ell ^\tau$ minimization, with $\tau$ larger than or equal to zero and less than or equal to 1 when the operator is not the identity, other techniques have recently been investigated. Here, we mention iteratively reweighted convex majorization algorithms [10], alternating direction method of multiplier (ADMM) [9] and finally a Newton-type solution algorithm for a regularized version of the original problem [6]. Finally we recall the paper [21], where a novel algorithm for nonsmooth nonconvex optimization with linear constraints is proposed, consisting of a generalization of the well-known nonstationary-augmented Lagrangian method for convex optimization. The convergence to critical points is proved and several tests were made for free-discontinuity variational models, such as the Mumford–Shah functional. The nonsmoothness considered in [21] does not allow singular behaviour of the type that the $\ell ^\tau$ term, with $\tau$ larger than or equal to zero and strictly less than 1 does.

The paper is structured as follows. In Sect. 2, Sect. 2.1, we state the precise assumptions, in Sect. 2.2, we prove existence for the problem in consideration, in Sect. 2.3, we propose the monotone scheme to solve a regularized version of the original problem and we prove its convergence, and finally in Sect. 2.4, we study the asymptotic behaviour as the concavity and regularization parameters go to zero. In Sect. 3, we present the precise form of our scheme. In Sect. 3.1, we discuss our numerical experience for cohesive evolution of fracture mechanics and in Sect. 3.2, we compare the performance of our scheme to that of GIST for three different test cases, the academical M-matrix example, an optimal control problem, and a microscopy imaging example.

## Existence and Monotone Algorithm

### Assumptions

We consider

1 $$\begin{aligned} \min _{x \in {\mathbb {R}}^n}J(x)=\frac{1}{2}|A x-b|_2^2+\sum _{i=1}^r\phi (\varLambda x)_i, \end{aligned}$$

where $$A \in \mathbb {M}^{m\times n}$$, $$\varLambda \in \mathbb {M}^{r\times n},$$$$b \in {\mathbb {R}}^m$$ and $$\phi (t): {\mathbb {R}}\rightarrow {\mathbb {R}}^+$$ satisfies

$$\begin{aligned} \mathbf{(H)} {\left\{ \begin{array}{ll} \text {(i)}\; &{}\phi \text{ is } \text{ even } \text{ with } \phi (0)=0, \text{ nondecreasing } \text{ for } t\ge 0 \text{ and } \text{ continuous };\\ \text {(ii)}\; &{}\phi \text{ is } \text{ differentiable } \text{ on } ]0,\infty [;\\ \text {(iii)}\; &{}\phi \text{ is } \text{ concave } \text{ on } {\mathbb {R}}^+;\\ \text {(iv)}\; &{} \text{ there } \text{ exists } \text{ a } \text{ neighbourhood } \text{ of } \text{ zero } \text{ where } \text{ the } \text{ function } t \rightarrow \frac{\phi '(t)}{t} \text{ is } \text{ monotone };\\ \end{array}\right. } \end{aligned}$$

Above monotonically increasing or decreasing are admitted. Throughout the rest of the paper, we will use the notation

$$\begin{aligned} \varPhi (\varLambda x):=\sum _{i=1}^r\phi (\varLambda x)_i. \end{aligned}$$

Under assumption $\mathbf{(H)}$, the following two cases are analysed:

-
  (i) $\phi (t)$ is a constant, when $|t|\ge t_0$ for some $t_0>0$;
  (ii) A is coercive, i.e. $\text{ rank }(A)=n$.
-
  (i) for some $\gamma >0$, it holds $\phi (at)=a^\gamma \phi (t)$ for all $t \in {\mathbb {R}}$ and $a \in {\mathbb {R}}^+$;
  (ii) $$\text{ Ker }(A)\cap \text{ Ker }(\varLambda )=\{0\}.$$

Three popular examples of nonconvex penalties which satisfy $\mathbf{(H)}$ and the assumptions on $\phi$ in (a) or (b) are the following:

$${{\varvec{\ell }}^\tau }$$
$$\tau \in ]0,1], \lambda >0$$
  2 $$\begin{aligned} \phi (t)=\lambda |t|^\tau , \end{aligned}$$
  satisfying (*b*)(*i*).
SCAD: $$\tau>1, \lambda >0$$
  3 $$\begin{aligned} \phi (t)=\left\{ \begin{array}{lll} \frac{\lambda ^2(\tau +1)}{2}, &{} \quad |t|\ge \lambda \tau ,\\ \frac{\lambda \tau |t|-\frac{1}{2}(t^2+\lambda ^2)}{\tau -1}, &{}\quad \lambda < |t|\le \lambda \tau ,\\ \lambda |t|, &{}\quad |t|\le \lambda , \end{array} \right. \, \end{aligned}$$
  satisfying (*a*)(*i*).
MCP: $$\tau>1, \lambda >0$$
  4 $$\begin{aligned} \phi (t)=\left\{ \begin{array}{lll} \lambda \left( |t|-\frac{t^2}{2\lambda \tau }\right) , &{}\quad |t|< \lambda \tau ,\\ \frac{\lambda ^2\tau }{2},&{}\quad |t|\ge \lambda \tau , \end{array} \right. \, \end{aligned}$$
  satisfying (*a*)(*i*).

#### Remark 2.1

The singularity at the origin of the three penalties leads to sparsity of the solution. In the SCAD and the MCP, the derivative vanishes for large values to ensure unbiasedness.

Problems as (1) with $\phi$ given by the $\ell ^\tau$-quasi-norm with $\tau \in ]0,1[$ were studied in [20]. For more details on its statistical properties, such as variable selection and oracle property, of the $\ell ^\tau$-quasi-norm, we refer to [14, 15, 27, 28].

The SCAD (smoothly clipped absolute deviation) ([11, 18]) has raised interest in relation to variable selection consistency and asymptotic estimation efficiency (see [18]). It can be obtained upon integration of the following formula for $\tau >2$

$$\begin{aligned} \phi (t)=\lambda \int _0^{|t|}\min \left( 1,\frac{\max (0,\lambda \tau -|s|)}{\lambda (\tau -1)}\right) \mathrm{d}s. \end{aligned}$$

The MCP (minimax concave penalty) [13] can be recovered from the following formula

$$\begin{aligned} \phi (t)=\lambda \int _0^{|t|} \max \left( 0,1-\frac{|s|}{\lambda \tau }\right) \mathrm{d}s. \end{aligned}$$

It minimizes the maximum concavity $\sup _{0<t_1<t_2}\frac{\left( \phi '(t_1)-\phi '(t_2)\right) }{(t_2-t_1)}$ subject to the constraints $\phi '(t)=0$ for any $|t|\ge \lambda \tau$ (unbiasedness) and $\phi '(0^{\pm })=\pm \lambda$ (feature selection). The condition $\tau >1$ ensures the wellposedness of the thresholding operator.

### Existence

First, we prove coercivity of the functional J in (1) under assumptions (a) or (b).

#### Lemma 2.1

Let assumptions **(H)** and either (a) or (b) hold. Then, the functional J in (1) is coercive.

#### Proof

Under assumption (a), the coercivity of J follows trivially. Suppose now that (b) holds. Then, the result follows by similar arguments to that used in [20], Theorem 1 (where $$\phi $$ is the $$\ell ^\tau $$ quasi-norm). We proceed by contradiction and we suppose that $$|x_k|_2\rightarrow + \infty $$ and $$J(x_k)$$ is bounded. For each *k*, let $$x_k=t_kz_k$$ be such that $$t_k\ge 0, x_k \in {\mathbb {R}}^n$$ and $$|z_k|_2=1. $$ By (b) (i), we have $$ \varPhi (\varLambda z_k)= \frac{1}{t_k^\gamma }\varPhi (\varLambda x_k) $$ and then since $$t_k \rightarrow + \infty $$ and $$J(x_k)$$ is bounded, we have for $$k \rightarrow + \infty $$

$$\begin{aligned} \displaystyle 0\le |A z_k|_2^2+ \varPhi (\varLambda z_k)=\frac{1}{ t_k^2}|A x_k|_2^2+ \frac{1}{t_k^\gamma }\varPhi (\varLambda x_k)\le \frac{1}{t_k^{\min \{2,\gamma \}}}\left( |A x_k|_2^2+\varPhi (\varLambda x_k)\right) \rightarrow 0 \end{aligned}$$

and hence $\lim _{k \rightarrow + \infty } |Az_k|_2^2+\varPhi (\varLambda z_k)=0.$ By compactness, the sequence $\{z_k\}$ has an accumulation point $\bar{z}$ such that $|\bar{z}|=1$ and $\bar{z} \in \text{ Ker }(A)\cap \text{ Ker }(\varLambda )$, which contradicts (b) (ii). $\square$

In the following theorem, we state the existence of at least a minimizer to (1) under either (a) or (b). We omit the proof since it follows directly by the continuity and coercivity of the functional in (1).

#### Theorem 2.1

Let assumptions **(H)** and either (a) or (b) hold. Then, there exists at least one minimizer to problem (1).

#### Remark 2.2

We remark that when assumption (a) (i) holds but *A* is not coercive, existence can still be proven in case $\varLambda \in {\mathbb {R}}^{n\times n}$ is invertible. Indeed, by the invertibility of $\varLambda$, one can define $\bar{y}=\varLambda ^{-1}{\bar{x}}$, where ${\bar{x}}$ is a minimizer of $\bar{J}(x)=\frac{1}{2}|(A\varLambda ^{-1})x-b|_2^2+\varPhi (x)$ and prove that $\bar{y}$ is a minimizer of (1). The existence of a minimizer for the functional $\bar{J}$ was proven in [26], Theorem 2.1.

However, in our analysis, we cover the two cases (a) and (b) since when (a) (ii) is replaced by the invertibility of $\varLambda$, we cannot prove the coercivity of *J*, which is a key element for the convergence of the algorithm that we analyse (see the following section).

### A Monotone Convergent Algorithm

Following [7], in order to overcome the singularity of the function $\phi (t)$ near $t=0$, we consider for $\varepsilon >0$ the following regularized version of (1)

5 $$\begin{aligned} \min _{x \in {\mathbb {R}}^n}J_\varepsilon (x) = \frac{1}{2}|A x-b|_2^2+ \varPsi _\varepsilon (|\varLambda x|_2^2), \end{aligned}$$

where for $$t \ge 0$$

6 $$\begin{aligned} \varPsi _{\varepsilon }(t)= \left\{ \begin{array}{ll} \frac{\phi '(\varepsilon )}{2\varepsilon }t+\left( 1-\frac{\phi '(\varepsilon ) \varepsilon }{2\phi (\varepsilon )}\right) \phi (\varepsilon ) &{}\quad \text{ for } \,\, 0\le t \le \varepsilon ^2\\ \phi (\sqrt{t}) &{}\quad \text{ for } \,\, t \ge \varepsilon ^2, \end{array} \right. \, \end{aligned}$$

and $\varPsi _\varepsilon (|\varLambda x|_2^2)$ is short for $\sum _{i=1}^r \varPsi _\varepsilon (|(\varLambda x)_i|^2)$. Note that

7 $$\begin{aligned} \varPsi '_\varepsilon (t)=\frac{1}{\max \left\{ \frac{2\varepsilon }{\phi '(\varepsilon )},\frac{2\sqrt{t}}{\phi '(\sqrt{t})}\right\} } > 0 \quad \text{ on } [0, \infty [, \end{aligned}$$

hence $\varPsi _\varepsilon$ is $C^1$ and by assumption **(H)** (iii) is concave on $[0,\infty [$. Moreover, we have also that the map $t \rightarrow \varPsi _\varepsilon (t^2) \in C^1(]-\infty , \infty [)$. Concerning the denominator in (7), let us point out that due to concavity of $\phi$ and **(H)**(i), the derivative $\phi '$ is non-increasing on $]0,\infty [$. Consequently $x\rightarrow \frac{\phi '(x)}{x}$ is non-increasing on $]0,\infty [$. Moreover, unless $\phi$ is identically 0, by **(H)** (i)–(iii), there exists an interval $]0, \eta [$ such that $\phi '(t)>0$ for $t \in ]0,\eta [$. It is always assumed that $\epsilon \in ]0, \eta [$.

The necessary optimality condition for (5) is given by

8 $$\begin{aligned} A^*A x+\varLambda ^* \frac{1}{\max \left\{ \frac{\varepsilon }{\phi '(\varepsilon )},\frac{|\varLambda x|_2}{\phi '(|\varLambda x|_2)}\right\} }\varLambda x=A^*b, \end{aligned}$$

the second addend is short for the vector with *l*-component $\sum _{i=1}^r(\varLambda ^*)_{li} \frac{1}{\max \left\{ \frac{\varepsilon }{\phi '(\varepsilon )},\frac{|(\varLambda x)_i|}{\phi '(|(\varLambda x)_i|)}\right\} }(\varLambda x)_i$. For convenience of exposition in the following, we write (8) in the more compact notation

$$\begin{aligned} A^*A x+2\varLambda ^* \varPsi '_\varepsilon (|\varLambda x|_2^2)\varLambda x=A^*b, \end{aligned}$$

where the *l*-component of the second addend is given by $\sum _{i=1}^r(\varLambda ^*)_{li} \varPsi '_\varepsilon (|(\varLambda x)^2_i|)(\varLambda x)_i$.

This can equivalently be expressed as

9 $$\begin{aligned} A^*A x+2\varLambda ^* \varPsi '_\varepsilon (|y|_2^2)y=A^*b \quad \text{ with } y=\varLambda x. \end{aligned}$$

In order to solve (9), the following iterative procedure is considered:

10 $$\begin{aligned} A^*A x^{k+1}+2\varLambda ^* \varPsi '_\varepsilon (|y^k|_2^2)y^{k+1}=A^*b \quad \text{ where } y^{k}=\varLambda x^{k}. \end{aligned}$$

Existence of a unique solution to (10) follows from (a) (ii), respectively, (b) (ii) and the fact that $\varPsi '_\varepsilon (t)>0$ for $t\in [0,\infty [$, where $\varPsi '_\varepsilon (0)$ is considered as derivative from the right. We have the following convergence result.

#### Theorem 2.2

Assume **(H)** and either (a) or (b). For $\varepsilon >0$, let $\{x_k\}$ be generated by (10). Then, $J_{\varepsilon }(x_k)$ is strictly monotonically decreasing, unless there exists some *k* such that $x^k = x^{k+1}$, and $x^k$ satisfies the necessary optimality condition (9). Moreover, every cluster point of $x^k$, of which there exists at least one, is a solution of (9).

#### Proof

The proof strongly depends on the coercivity of the functional *J* and it follows arguments similar to those of [7, Theorem 4.1].

Multiplying (10) by $x^{k+1}-x^k$, we get

11 $$\begin{aligned}&\frac{1}{2}|A x^{k+1}|_2^2-\frac{1}{2}|A x^k|_2^2+\frac{1}{2}|A (x^{k+1}-x^{k})|_2^2+ \left( 2\varPsi '_\varepsilon (|y^k|_2^2)y^{k+1},y^{k+1}-y^{k}\right) \nonumber \\&\quad =(A^*b,x^{k+1}-x^k). \end{aligned}$$

Note that for each $i=1, \ldots , n$, we have

12 $$\begin{aligned} y_i^{k+1}\left( y_i^{k+1}-y_i^k\right) =\frac{1}{2}\left( |y_i^{k+1}|^2-|y_i^{k}|^2+|y_i^{k+1}-y_i^{k}|^2\right) . \end{aligned}$$

By assumption $\mathbf{(H)}\,(iii)$, the function $t \rightarrow \varPsi _\varepsilon (t)$ is concave on $[0,\infty )$, and thus

13 $$\begin{aligned} 2\varPsi _\varepsilon (|y_i^{k+1}|^2)-2\varPsi _\varepsilon (|y_i^{k}|^2)-\varPsi '_\varepsilon (|y_i^k|^2)(|y_i^{k+1}|^2-|y_i^{k}|^2) \le 0. \end{aligned}$$

Using (12) and (13), we obtain the estimate

14 $$\begin{aligned}&(2 \varPsi '_\varepsilon (|y^{k}|^2) y^{k+1}, y^{k+1}-y^k) \nonumber \\&\quad =\sum _i \varPsi '_\varepsilon (|y_i^k|^2)(|y_i^{k+1}-y_i^k|^2+|y_i^{k+1}|^2-|y_i^{k}|^2)\nonumber \\&\quad \ge \sum _i \varPsi '_\varepsilon (|y_i^k|^2)(|y_i^{k+1}-y_i^k|^2)+2\varPsi _\varepsilon (|y_i^{k+1}|^2)-2\varPsi _\varepsilon (|y_i^k|^2). \end{aligned}$$

Then, using (11), (14) and the definition of $J_\varepsilon$, we get

15 $$\begin{aligned} J_\varepsilon (x^{k+1})+\frac{1}{2}|A(x^{k+1}-x^k)|_2^2+\sum _i\varPsi '_\varepsilon (|y_i^k|^2)|y_i^{k+1}-y_i^{k}|^2 \le J_\varepsilon (x^k). \end{aligned}$$

From (15) and the coercivity of $J_\varepsilon$, it follows that $\{x^k\}_{k=1}^\infty$ and thus $\{y^{k}\}_{k=1}^\infty$ are bounded. Consequently, from (15) and (7), there exists a constant $\kappa >0$ such that

16 $$\begin{aligned} J_\varepsilon (x^{k+1}) +\frac{1}{2}|A(x^{k+1}-x^k)|_2^2+\kappa |y^{k+1}-y^{k}|_2^2 \le J_\varepsilon (x^k). \end{aligned}$$

Conditions (a) (ii), (b) (ii), respectively, imply that $J_\varepsilon (x_k)$ is strictly decreasing unless $x^k=x^{k+1}$. In the latter case, from (10), we infer that $x^k$ solves (9), from which we conclude the first part of the theorem.

From (16), we conclude that

17 $$\begin{aligned} \sum _{k=0}^\infty |A(x^{k+1}-x^k)|_2^2+\kappa |y^{k+1}-y^{k}|_2^2 <\infty . \end{aligned}$$

Since $\{x^k\}_{k=1}^\infty$ is bounded, there exists a subsequence and ${\bar{x}} \in {\mathbb {R}}^n$ such that $x^{k_l}\rightarrow {\bar{x}}$. By (17), we get

$$\begin{aligned} \lim _{k\rightarrow \infty }|A(x^{k+1}-x^k)|_2^2+\kappa |y^{k+1}-y^{k}|_2^2=0. \end{aligned}$$

Then, by using the coercivity of A under assumption (a) and the fact that $\text{ Ker }(A)\cap \text{ Ker }(\varLambda )=\{0\}$ under assumption (b), we conclude that $\lim _{k\rightarrow \infty }(x^{k+1}-x^{k})=0$ and hence $x^{k_l+1} \rightarrow {\bar{x}}$. We can now pass to the limit with respect to *k* in (10), to obtain that ${\bar{x}}$ is a solution to (9). $\square$

In the following proposition, we establish the convergence of (5) to (1) as $\varepsilon$ goes to zero.

#### Proposition 2.1

Assume **(H)** and either (a) or (b). Denote by $\{x_\varepsilon \}_{\varepsilon >0}$ a solution to (5). Then any cluster point of $\{x_\varepsilon \}_{\varepsilon >0}$, of which there exists at least one, is a solution of (1).

#### Proof

From the coercivity of $J_\varepsilon$, we have that $\{x_\varepsilon \}_{\varepsilon }$ is bounded for $\varepsilon$ small. Hence, there exists a subsequence and ${\bar{x}} \in {\mathbb {R}}^n$ such that $x_{\varepsilon _l} \rightarrow {\bar{x}}$ as $l \rightarrow \infty$.

By property **(H)** (i) of $\phi$, we have

18 $$\begin{aligned} \lim _{t\rightarrow 0 }\phi (t)=0 \quad \text{ and } \quad \phi '(t)\ge 0 \quad \forall t \ge 0. \end{aligned}$$

By the concavity of the function $\phi$, we have $\phi (t)-\phi (s)\le \phi '(s)(t-s)$ for $s \in ]0,\infty [, t \in [0,\infty [,$ and, by choosing $s=\varepsilon$ and $t=0$ and by (18), we get for $\varepsilon$ small enough

19 $$\begin{aligned} \phi '(\varepsilon )\varepsilon \rightarrow 0 \,\, \text{ as } \varepsilon \rightarrow 0. \end{aligned}$$

By the definition of $\varPsi _\varepsilon$, (18) and (19), we obtain that $\varPsi _\varepsilon (t)$ converges uniformly to $\phi (\sqrt{t})$ as $\varepsilon \rightarrow 0$, equivalently

$$\begin{aligned} \sup _{t\in [0,\infty [}\left| \varPsi _\varepsilon (t) -\phi (\sqrt{t})\right| \rightarrow 0\quad \text{ as } \varepsilon \rightarrow 0, \end{aligned}$$

from which we obtain

20 $$\begin{aligned} \varPsi _{\varepsilon _l}(|\varLambda x_{\varepsilon _l}|^2)=\sum _{i=1}^r\varPsi _\varepsilon (|(\varLambda x_{\varepsilon _l})_i|^2) \rightarrow \sum _{i=1}^r \phi (\varLambda x_{\varepsilon _l})_i=\varPhi (\varLambda {\bar{x}})\quad \text{ as } l \rightarrow \infty . \end{aligned}$$

Since $x_{\varepsilon _l}$ solves (5) for $\varepsilon =\varepsilon _l$, by letting $l \rightarrow \infty$ and using (20), we easily get that ${\bar{x}}$ is a solution of (1). $\square$

### Asymptotic Behaviour as $\lambda \searrow 0$ and $\tau \searrow 0$ for the Power Law

We discuss the asymptotics as $\lambda$ and $\tau$ go to zero in (1) for $\phi (t)=|t|^\tau , \tau \in ]0,1[$, which we repeat for convenience

21 $$\begin{aligned} \min _{x \in {\mathbb {R}}^n}\frac{1}{2}|A x-b|_2^2+\lambda |\varLambda x|_\tau ^\tau , \end{aligned}$$

where $A, b, \varLambda$ are as in (1), $\tau \in ]0,1[, \lambda >0$ and $|\varLambda x|_\tau ^\tau =\sum _{i=1}^r |(\varLambda x)_i|^\tau .$ First, we analyse the convergence as $\lambda \rightarrow 0$ for any fixed $\tau >0$. We denote by *P* the orthogonal projection of ${\mathbb {R}}^m$ onto $\text{ Ker }(A^{*})$ and set $\tilde{b} = (I-P)b \in \text{ Rg }(A)$. Then, $|Ax -b|_2^2=|Ax-\tilde{b}|_2^2+|Pb|_2^2.$ For $\tau >0$ fixed, consider the problem

22 $$\begin{aligned} \min _{x} | \varLambda x|_\tau ^\tau \quad \text { subject to } Ax ={\tilde{b}}. \end{aligned}$$

#### Theorem 2.3

Assume that $ker(A) \cap ker (\varLambda )=\{0\}$ and let $\tau >0$ be fixed. For each $\lambda >0$, let $x_{\lambda }$ be a minimizer of (21). Then, every cluster point of $\{x_\lambda \}$, of which there exists at least one, is a solution to (22).

#### Proof

Let ${\tilde{x}} \in \mathbb {R}^n$ be an arbitrary solution to $A x - {\tilde{b}} =0$. By optimality of $x_\lambda$, we have

23 $$\begin{aligned} \frac{1}{2}|Ax_{\lambda } - \tilde{b}|_2^2+\lambda |\varLambda x_\lambda |_\tau ^\tau \le \frac{1}{2}|A\tilde{x}-\tilde{b}|_2^2+\lambda |\varLambda \tilde{x}|_\tau ^\tau =\lambda |\varLambda \tilde{x}|_\tau ^\tau . \end{aligned}$$

We conclude that

24 $$\begin{aligned} \lim _{\lambda \rightarrow 0} |Ax_{\lambda } -\tilde{b}|_2^2=0, \quad \text { and }|\varLambda x_\lambda |^\tau _\tau \le |\varLambda {\tilde{x}}|^\tau _\tau \text { for all } {\tilde{x}} \text { satisfying } Ax = {\tilde{b}}. \end{aligned}$$

In particular, the families $\{A x_\lambda \}$ and $\{\varLambda x_\lambda \}$ are bounded in $\lambda$. Since by assumption we have $ker(A) \cap ker (\varLambda )=\{0\}$, it follows that $\{ x_\lambda \}$ is bounded. Hence, there exists a convergent subsequence $x_{\lambda _\ell }$ with some limit ${\bar{x}}$. From (24), it follows that ${\bar{x}}$ is a solution to (22). $\square$

Now, we prove the convergence as $\tau \rightarrow 0$ for any fixed $\lambda >0$ of (21) to the related $\ell ^0$-problem

25 $$\begin{aligned} \min _{x \in {\mathbb {R}}^n}\frac{1}{2}|Ax-b|_2^2+\lambda |\varLambda x|_0, \end{aligned}$$

where for any $$x \in {\mathbb {R}}^n$$$$ |x|_0=\sum _{k=1}^n|x_k|^0= \text{ number } \text{ of } \text{ nonzero } \text{ elements } \text{ of } x. $$ The precise statement is given in the following theorem.

#### Theorem 2.4

Assume that $\text{ rank }(A)=n$, and that $\varLambda \in {\mathbb {R}}^{n \times n}$ is regular, and let $\lambda >0$ be fixed. Then, every cluster point (of which there exists at least one) of solutions $\{x_{\tau }\}$ to (21) converges as $\tau \searrow 0$ to a solution of (25).

#### Proof

By Theorem 2.1, there exists a global solution $x_\tau$ for each $\tau >0$. Since

$$\begin{aligned} \frac{1}{2}|A x_\tau -b|_2^2 + \lambda |\varLambda x_\tau |_\tau ^\tau \le \frac{1}{2}|b|_2^2 \end{aligned}$$

we have that $\{A x_\tau \}_{\tau >0}$ is bounded. Now, $\text{ ker }A=\{0\}$ implies the existence of a subsequence, denoted by the same symbols, and ${\bar{x}} \in {\mathbb {R}}^n$, such that $x_\tau \rightarrow {\bar{x}}$ for $\tau \searrow 0$.

For any fixed $i \in \{1,\ldots , r\}$, denote $y_{\tau }=|(\varLambda x_{\tau })_i|$ and ${\bar{y}}=|(\varLambda {\bar{x}})_i|$ and notice that $y_{\tau } \rightarrow \bar{y}$ as $\tau \rightarrow 0$. If ${\bar{y}}>0$, we have $\log (y_\tau ^\tau )=\tau \log (y_\tau ) \rightarrow 0$ as $\tau \rightarrow 0$ and thus $y_\tau ^\tau \rightarrow 1$ as $\tau \rightarrow 0.$

Next, we assume that ${\bar{y}}=0$. We claim that there exists $\bar{\tau } >0$, such that $y_\tau =0$ for all $\tau \in ]0,\bar{\tau }[$. Arguing by contradiction, assume that there exists a subsequence, denoted by the same symbols again, such that $y_\tau \rightarrow {\bar{y}}=0$ for $\tau \searrow 0$ and $y_\tau >0$ for all $\tau$ sufficiently small. From [20], Corollary 1, we know that

$$\begin{aligned} |y_\tau |\ge \left( \frac{2\beta (1-\tau )}{|(A\varLambda ^{-1})_i|_2^2}\right) ^{\frac{1}{2-\tau }}, \end{aligned}$$

which, by taking the limit $\tau \searrow 0$, implies

$$\begin{aligned} 0=|{\bar{y}}|\ge \left( \frac{2\beta }{|(A\varLambda ^{-1})_i|_2^2}\right) ^{\frac{1}{2}}. \end{aligned}$$

Since $\varLambda$ is regular and $\text{ rank }(A)=n$, we have $|(A\varLambda ^{-1})_i|_2^2 \ne 0$ and we obtained the desired contradiction.

By using the above arguments for all $i=1,\ldots , r$, we have $|(\varLambda x_\tau )_i|^\tau \rightarrow |(\varLambda {\bar{x}})_i|^0$ as $\tau \rightarrow 0$, and then we conclude

26 $$\begin{aligned} |\varLambda x_\tau |^{\tau }_\tau \rightarrow |\varLambda {\bar{x}}|_0 \quad \text{ as } \tau \rightarrow 0. \end{aligned}$$

By the optimality of $x_\tau$, we get $\frac{1}{2}|Ax_\tau -b|_2^2+\lambda |\varLambda x_\tau |^{\tau }_\tau \le \frac{1}{2}|Ax-b|_2^2+\lambda |\varLambda x|_\tau ^\tau ,$ for all $x \in {\mathbb {R}}^n.$ Then, the proof follows by taking the limit $\tau \rightarrow 0$ and using (26) to obtain

$$\begin{aligned} \frac{1}{2}|A{\bar{x}}-b|_2^2+\lambda |\varLambda {\bar{x}}|_0\le \frac{1}{2}|Ax-b|_2^2+\lambda |\varLambda x|_0, \quad \text{ for } \text{ all } x \in {\mathbb {R}}^n. \end{aligned}$$

$$\square $$

#### Remark 2.3

The assumption on $\ker (A)$ is caused by the fact that the $|\cdot |_0$ functional is not radially unbounded on $\mathbb {R}^n$. Since Theorem 2.4 provides an existence result for (25), such an assumption is natural. To the best of our knowledge, existence of minimizers of (25) has only been addressed under assumption (a) (ii) (or assumptions implying it). In the context of algorithm development further, $\ell ^1$- or $\ell ^2$- regularization is often added, we refer to [12, 29, 30].

## Algorithm and Numerical Results

For convenience, we recall the algorithm in the following form.
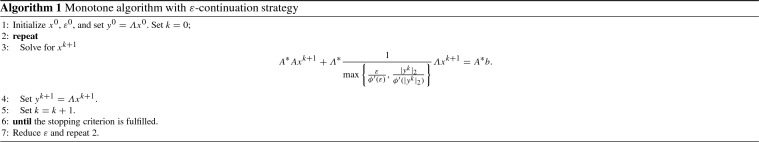


### Remark 3.1

Note that an $\varepsilon$-continuation strategy is performed, that is, the procedure is performed for an initial value $\varepsilon ^0$ and then $\varepsilon$ is decreased up to a certain value. More specifically, in all our experiments, $\varepsilon$ is initialized with $10^{-1}$ and decreased up to $10^{-12}$.

### Remark 3.2

The stopping criterion is based on the $l^\infty$-norm of Eq. (9) and the tolerance is set to $10^{-3}$ in all the following examples, except for the fracture problem where it is of the order of $10^{-15}$.

In the following subsection, we present our numerical results in cohesive fracture mechanics. Then, in Sect. 3.2, the performance of our algorithm is compared to two other schemes for nonconvex and nonsmooth optimization problems.

### Application to Quasi-Static Evolution of Cohesive Fracture Models

In this section, we focus on the numerical realization of quasi-static evolutions of cohesive fractures. These kinds of problems require the minimization of an energy functional, which has two components: the elastic energy and the cohesive fracture energy. The underlying idea is that the fracture energy is released gradually with the growth of the crack opening. The cohesive energy, denoted by $\theta$, is assumed to be a monotonic non-decreasing function of the jump amplitude of the displacement, denoted by $\llbracket u \rrbracket$. Cohesive energies were introduced independently by Dugdale [31] and Barenblatt [32]; we refer to [19] for more details on the models. Among the vast existing literature on fracture mechanics, we also point out [33–35], as some of the most significant references to us. Let us just remark that the two models differ mainly in the evolution of the derivative $\theta '(\llbracket u\rrbracket )$, that is, the *bridging force*, across a crack amplitude $\llbracket u \rrbracket$. In Dugdale’s model, this force keeps a constant value up to a critical value of the crack opening and then drops to zero. In Barenblatt’s model, the dependence of the force on $\llbracket u \rrbracket$ is continuous and decreasing.

In this section, we test the $\ell ^\tau$-term $0<\tau <1$ as a model for the cohesive energy. In particular, the cohesive energy is not differentiable in zero and the bridging force goes to infinity when the jump amplitude goes to zero. Note also that the bridging force goes to zero when the jump amplitude goes to infinity.

We denote by $u\,:\, \varOmega \rightarrow {\mathbb {R}}$ the displacement function. The deformation of the domain is given by an external force which we express in terms of an external displacement function $g\,:\,\varOmega \times [0,T] \rightarrow {\mathbb {R}}$. We require that the displacement *u* coincides with the external deformation, that is, $u|_{\partial \varOmega }=g|_{\partial \varOmega }.$ We denote by $\varGamma$ the point of the (potential) crack, and by $\theta (\llbracket u \rrbracket )_\varGamma$ the value of the cohesive energy $\theta$ on the crack amplitude of the displacement $\llbracket u \rrbracket$ on $\varGamma$. Since we are in a quasi-static setting, we introduce the time discretization $0=t_0<t_1< \cdots <t_T=T$ and look for the equilibrium configurations which are minimizers of the energy of the system. This means that for each $i \in \{0, \ldots , T\}$ we need to minimize the energy of the system

$$\begin{aligned} J(u)=\frac{1}{2}\int _{\varOmega \backslash \varGamma }|a(x)\nabla u|_2^2 \mathrm{d}x +\theta (\llbracket u \rrbracket )_\varGamma \end{aligned}$$

with respect to a given boundary datum *g*: $u^*\in \mathop {{\mathrm{argmin}}}\limits _{u=g(t_i) \text{ on } \partial \varOmega } J(u).$ The function $a(\cdot )$ measures the degree of homogeneity of the material, e.g. $a(x)\equiv 1$ means that the material is homogeneous.

In our experiments, we consider three different types of cohesive energy, the $$\ell ^\tau $$$$\tau \in ]0,1[$$, SCAD, and MCP penalties as defined in (2), (3), (4), respectively.

In Sects. 3.1.1 and 3.1.2, we show our results for one-dimensional and two-dimensional experiments, respectively.

#### One-Dimensional Experiments

We consider the one-dimensional domain $\varOmega =[0,1]$ and we chose the point of crack as the midpoint $\varGamma =0.5$. We divide $\varOmega$ into 2*N* intervals and approximate the displacement function with a function $u_h$ that is piecewise linear on $\varOmega \backslash \varGamma$ and has two degrees of freedom on $\varGamma$ to represent correctly the two lips of the fracture, denoting with $u_{N}^{-}$ the one on [0, 0.5] and $u_{N}^{+}$ the one on [0.5, 1]. We discretize the problem in the following way

27 $$\begin{aligned} J_h(u_h)=\frac{1}{2} \sum _{i=1}^{2N} 2N |a_i(u_i -u_{i-1})|^2+ \theta (\llbracket u_N \rrbracket ), \end{aligned}$$

where, if $i\le N$, we identify $u_N=u_N^-$, while for $i>N, u_N=u_N^+$ and $a_i$ denotes the piecewise linear approximation of the material inhomogeneity function. We remark that the jump of the displacement is not taken into account in the sum, and the gradient of *u* is approximated with finite difference of first order. The Dirichlet condition is applied on $\partial \varOmega =\{0,2l\}$ and the external displacement is chosen as $u(0,t)=0, u(2l,t)=2lt.$ To enforce the boundary condition in the minimization process, we add it to the energy functional as a penalization term. Hence, we solve the following unconstrained minimization problem

28 $$\begin{aligned} \min N|A u_h -b|_2^2+\theta (\llbracket u_N \rrbracket ), \end{aligned}$$

where the operator $A \in {\mathbb {R}}^{(2N+1)\times (2N+1)}$ is given by $A=RD$ where $R\in {\mathbb {R}}^{(2N+1)\times (2N+1)}$ is the diagonal operator with *i*-entries $R_{ii}=a_i$ and $A=\left[ {\bar{D}}', [0\, \,\ldots \,\, 0 \, \,\gamma ]' \right] '.$ Here, ${\bar{D}} \in {\mathbb {R}}^{2N\times (2N+1)}$ is the backward finite difference operator *D* without the $N+1$ row, where we use the notation $D:=diag(-ones(2N,1),-1))+diag(ones(2N+1,1))\, : \,{\mathbb {R}}^{2N+1} \rightarrow {\mathbb {R}}^{2N+1}$. Moreover, $b \in {\mathbb {R}}^{2N+1}$ in (28) is given by $b=(0,\ldots , \gamma t_i)'$ and $\gamma$ is the penalization parameter. To compute the jump between the two lips of the fracture, we introduce the operator $D_f:{\mathbb {R}}^{2N+1} \rightarrow {\mathbb {R}}$ defined as $D_f=(0,\ldots , -1,1,0,\ldots ,0)$ where $-1$ and 1 are, respectively, in the *N* and $N+1$ positions. Then, we write the functional (28) as follows

29 $$\begin{aligned} \min N|A u_h -b|_2^2+ \theta ( D_fu). \end{aligned}$$

We consider the three different penalizations given by the $\ell ^\tau , \tau \in ]0,1[$, the SCAD, and the MCP penalties. Note that $\text{ KerA } =0$, hence assumptions (*a*) (*ii*) and (*c*)(*ii*) are satisfied and existence of a minimizer for (29) is guaranteed.

Our numerical experiments were conducted with a discretization in 2*N* intervals, $N=100$. The time step, in the time discretization of [0, *T*], with $T=3$, is set to $\mathrm{d}t=0.01$. The parameters of the energy functional $J_h(u_h)$ are set to $\lambda =1, \gamma =50$.

**Fig. 1:**
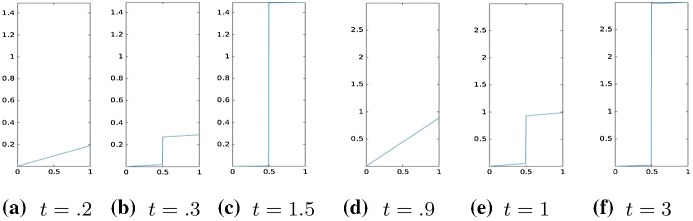
Three time-step evolution of the displacement for $\tau =.01$, $t = .2, .3, 1.5$ (left), $\tau =.1$, $t=.9, 1, 3$ (right). Results obtained by Algorithm 1

**Fig. 2:**
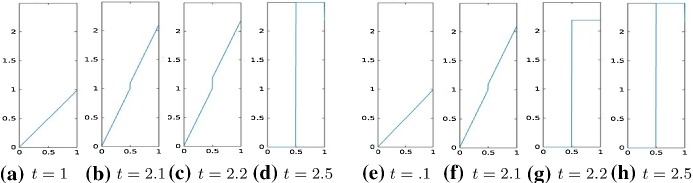
Four time-step evolution of the displacement for the SCAD model, $\tau =20$, $t=1, 2.1, 2.2, 2.5$ (left), $\tau =10$, $t = .1, 2.1, 2.2, 2.5$ (right). Results obtained by Algorithm 1

We remark that in the following experiments, the material function *a*(*x*) was always chosen as the identity. For tests with more general *a*(*x*), we refer to the two-dimensional experiments reported in the following subsection. In Figs. 1 and 2, we report our results obtained by Algorithm 1, respectively, for the models $\ell ^p$ and SCAD. In each figure, we show time frames to represent the evolution of the crack for different values of the parameter $\tau$. Each time frame consists of three different time steps $(t_1, t_2, t_3)$, where $t_2, t_3$ are chosen as the first instant where the prefracture and the fracture appear.

We observe the three phases that we expect from a cohesive fracture model:

- *Pure elastic deformation*: in this case, the jump amplitude is zero and the gradient of the displacement is constant in $\varOmega \backslash \varGamma$;
- *Prefracture*: the two lips of the fracture do not touch each other, but they are not free to move. The elastic energy is still present.
- *Fracture*: the two parts are free to move. In this final phase, the gradient of the displacement (and then the elastic energy) is zero.

We point out that the model we consider describes a fracture process in which the crack amplitude $\llbracket u \rrbracket$ is allowed to differ from zero. In particular, there is no possibility of breaking of the material with the crack surfaces remaining in contact.

We remark that the formation of the crack is anticipated for smaller values of $\tau$. As we see in Fig. 1, for $\tau =.01$, prefracture and fracture are reached at $t=.3$ and $t=1.5$, respectively. As $\tau$ is increased to $\tau =.1$, prefracture and fracture occur at $t=1$ and $t=3$, respectively. We observe the same phenomenon for the SCAD (see Fig. 2).

We tested our algorithm also for the MCP model, where no prefracture phase can be observed, that is, the displacement breaks almost instantaneously to reach the complete fracture.

Finally, we remark that in our experiments, the residue is $O(10^{-16})$ and the number of iterations is small, e.g. 12, 15 for $\tau =.01, .1$, respectively.

#### Two-Dimensional Experiments

We consider the two-dimensional domain $\varOmega =]0,1[\times ]0,1]$ and we chose the one-dimensional subdomain $0.5\times ]0,1[$ as the line of crack. We proceed in the discretization of the problem analogously as in Sect. 3.1.1, that is, we divide [0, 1] into 2*N* intervals and approximate the displacement function with a function $u_h$, that is, piecewise linear in $\varOmega \backslash \varGamma$ and has two degrees of freedom on $\varGamma$ to represent correctly the two lips of the fracture. Define the operator $A \in {\mathbb {R}}^{3m(m-1)\times m^2}$ by $A=\left[ (R^1G_1)', (R^2D_2)', \gamma I_{m^2}\right] ',$ where $m=2N+2$ and $R^1 \in {\mathbb {R}}^{(m(m-1))\times m(m-1)}$ and $R^2 \in {\mathbb {R}}^{m(m-2)\times m(m-2)}$ are two diagonal operators approximating the degree of homogeneity of the material, $D_2\in {\mathbb {R}}^{m(m-2)\times m^2}$ is defined as

$$\begin{aligned} D_2=G_2(mN+1:mN+m,:)=[\quad ], \end{aligned}$$

where $G_1, G_2 \in {\mathbb {R}}^{m(m-1)\times m^2}$ are defined as follows $G_1=kron(I_m,D), \quad G_2=kron(D,I_m)$ and $D=:diag(-ones(m,1))+diag(ones(m-1,1),1) \in {\mathbb {R}}^{(m-1)\times m}$ without the last row. Again, we enforce the boundary condition by adding it to the energy functional as a penalization term. Hence, we solve the following unconstrained minimization problem

30 $$\begin{aligned} \min |A u_h -b|_2^2+\theta (D_f u), \end{aligned}$$

where $b \in {\mathbb {R}}^{3m(m-1)}$ in (30) is given by $b=(0,\ldots , \gamma g(t_i))'$, $g(t_i)$ is the discretization of the boundary datum *g* at time $t_i$ and $\gamma$ is the penalization parameter. Moreover, the jump of the crack is represented by the operator $D_f:=[0_{m,mN}, -I_m, I_m, 0_{m,m^2-mN-2m}]\in {\mathbb {R}}^{m\times m^2}$, where by $0_{r,s}$ we denote the null matrix of dimension $r\times s$.

**Fig. 3:**
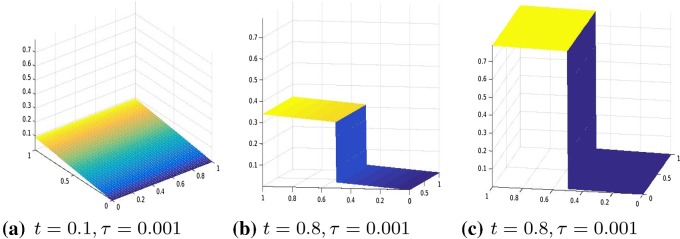
Displacement, $\theta (\cdot )=|\cdot |_\tau ^\tau$, with $\tau =0.001$, $R^1=R^2=I$, and boundary datum $g=g_1$

**Fig. 4:**
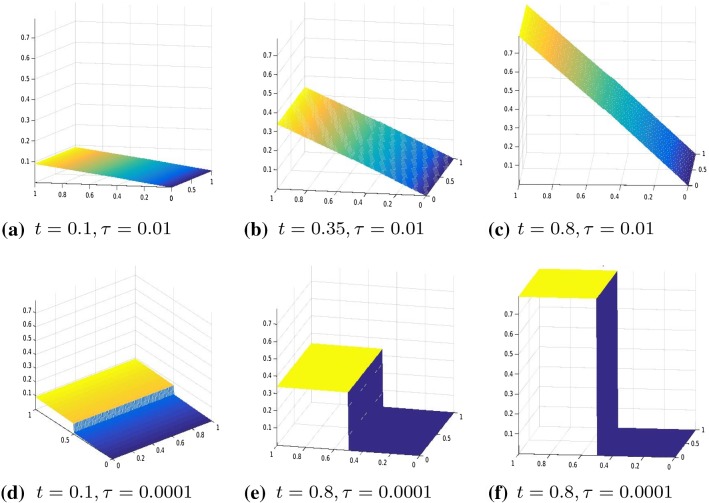
Displacement, $\theta (\cdot )=|\cdot |_\tau ^\tau$, comparison between $\tau =0.01$ and $\tau =0.0001$, $R^1=R^2=I$, boundary datum $g=g_1$

**Fig. 5:**
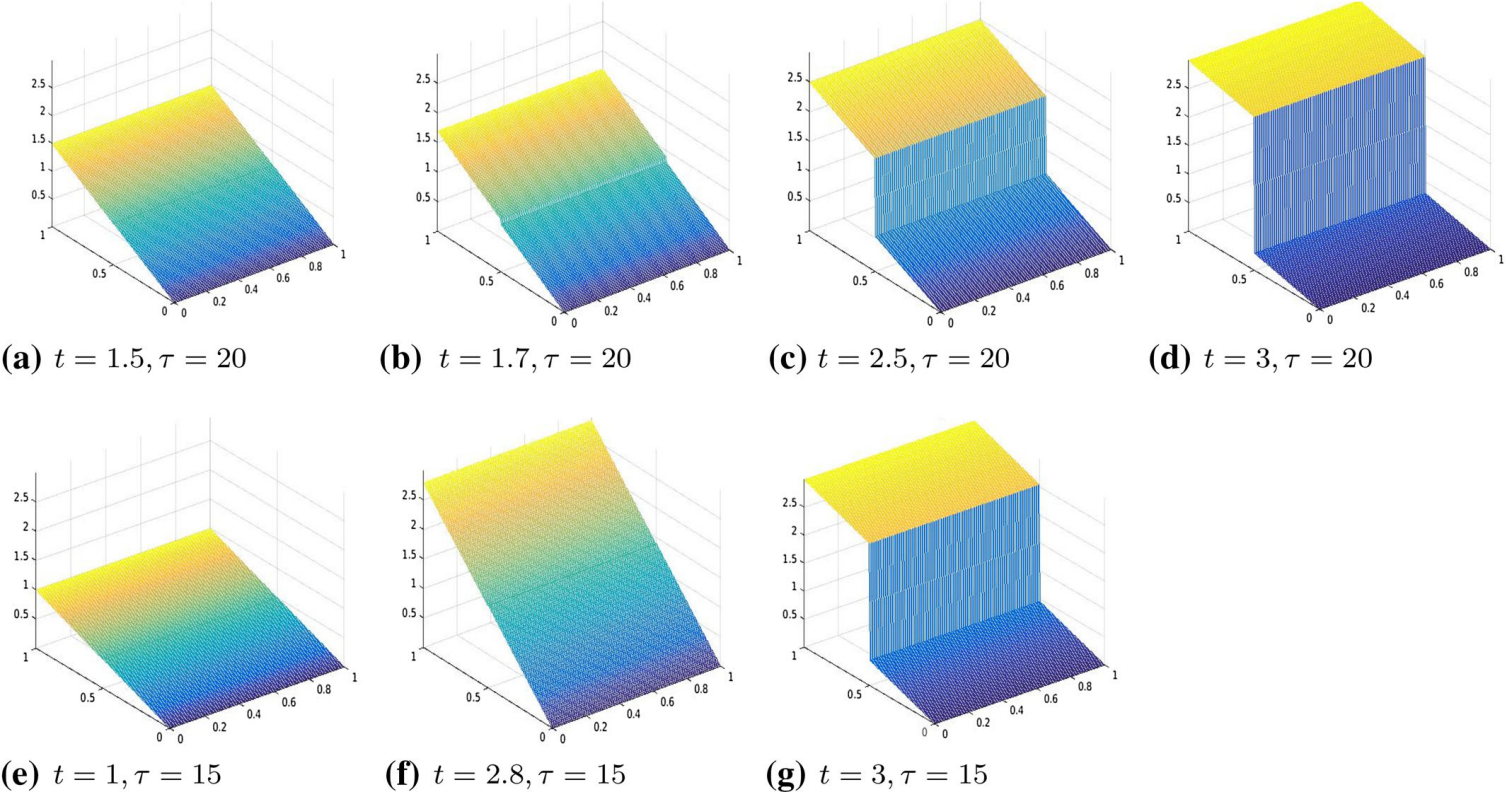
Displacement for the $\text{ SCAD }$ model, comparison between $\tau =20$ and $\tau =15$, $R^1=R^2=I$, boundary datum $g=g_1$

**Fig. 6:**
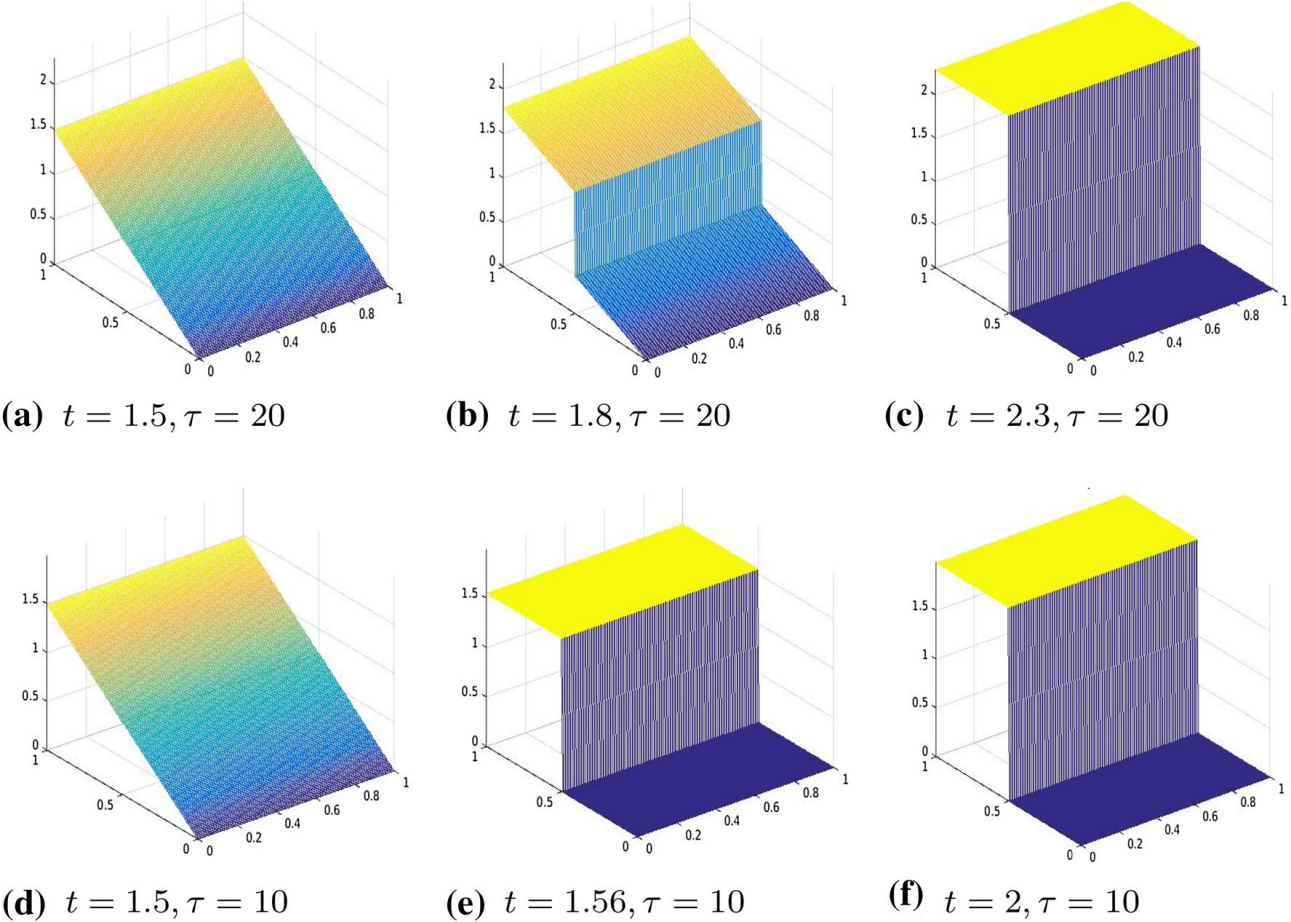
Displacement for the MCP model, comparison between $\tau =20$ and $\tau =10$, $R^1=R^2=I$, boundary datum $g=g_1$

**Fig. 7:**
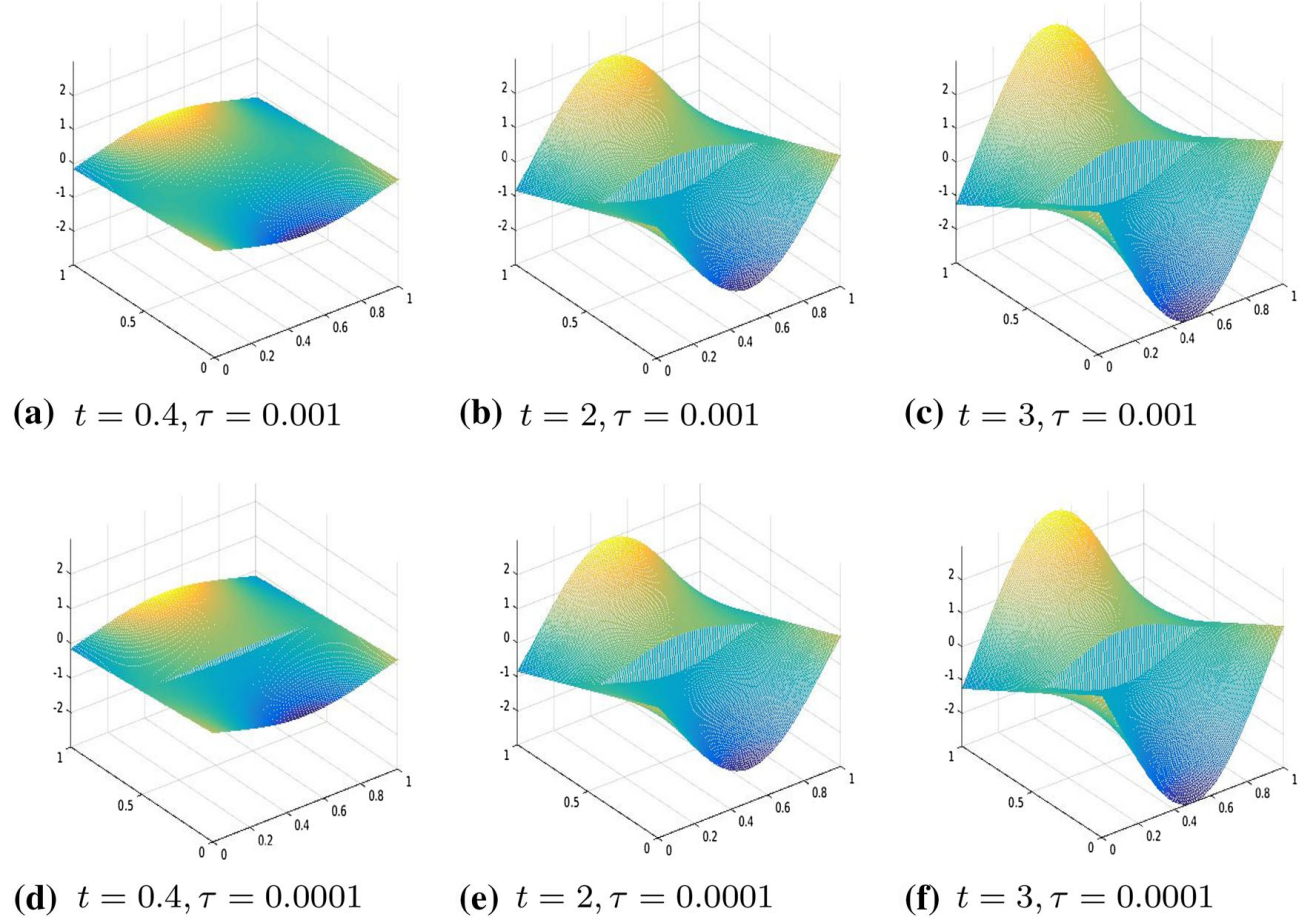
Displacement, $\theta (\cdot )=|\cdot |_\tau ^\tau$, comparison between $\tau =0.001$ and $\tau =0.0001$, $R^1=R^2=I$, boundary datum $g=g_2$

Our numerical experiments were conducted with a discretization in 2*N* intervals with $N=80$. The time step, in the time discretization of [0, *T*] with $T=3$, is set to $\mathrm{d}t=0.01$. The parameters of the energy functional $J_h(u_h)$ are set to $\lambda =1, \gamma =50$. We perform two different series of experiments with boundary data, respectively, resulting from evaluating $g_1, g_2$ on $\partial \varOmega$, where $g_1(t)(x)=(2x_1-0.5)t$ for every $t \in [0,1], x=(x_1,x_2) \in \varOmega$ and the other one with boundary datum $g_2(t)(x)=2t \cos (4(x_2-0.5))(x_1-0.5)$ for every $t \in [0,1], x=(x_1,x_2) \in \varOmega .$ In Figs. 3, 4, 5, and 6, we show the results obtained with boundary datum $g_1$ for each of the considered models, that is, $\ell ^\tau$, SCAD, and MCP and in Fig. 7, the ones with boundary datum $g_2$ for the $\ell ^\tau$ model. In the case of boundary datum $g_2$, we tested our algorithm also on the SCAD and the MCP models, obtaining similar results to the ones shown in Fig. 7. In these first experiments, the diagonal operators $R_1, R_2$ are taken as the identity, that is, we suppose to have an homogeneous material.

As expected from a cohesive fracture model, we observe the three phases of pure elastic deformation, prefracture, and fracture (see Sect. 3.1.1 for an explanation of the model and the three phases).

Also, prefracture and fracture are reached at different times for different values of $\tau$, typically they are anticipated for smaller values of $\tau$.

When the boundary datum is $g_2$, that is, not constant in the *y* direction, we note that the fracture is reached before in the part of the fracture line corresponding to the part of the boundary where the datum is bigger.

**Fig. 8:**
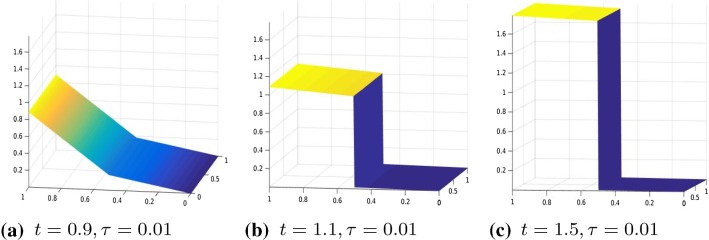
Displacement, $\theta (\cdot )=|\cdot |_\tau ^\tau$, $\tau =0.01$, $R^1, R^2$ given by (31)–(32), boundary datum $g=g_1$

**Fig. 9:**
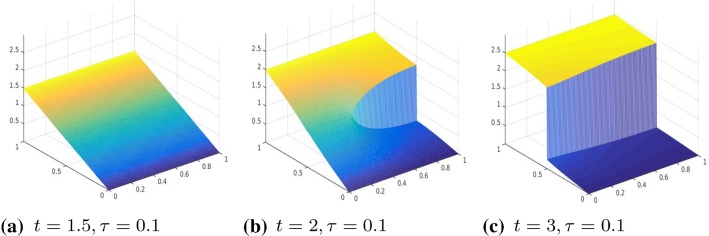
Displacement, $\theta (\cdot )=|\cdot |_\tau ^\tau$, $\tau =0.01, R^1,R^2$ given by (33), boundary datum $g=g_1$

**Fig. 10:**
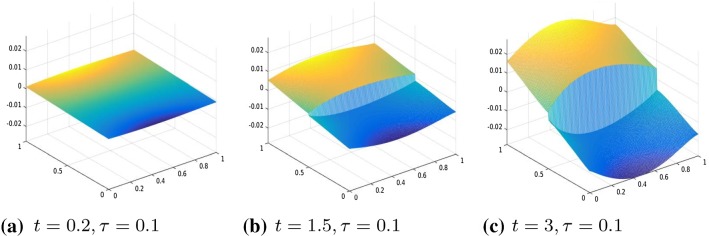
Displacement, $\theta (\cdot )=|\cdot |_\tau ^\tau$, $\tau =0.1$, $R^1,R^2$ given by (33), boundary datum $g=g_3$

In Figs. 8, 9, and 10, we tested the algorithm in case of a non-homogeneous material. In Fig. 8, we show the result for a two-material specimen, that is, we took

31 $$\begin{aligned}&\begin{array}{lll} R^1_{ii}=600 &{} \quad i=1, \ldots , (m-1)(N+1), \\ R^1_{ii}=1 &{} \quad i=(m-1)(N+1)+1, \ldots , 2(m-1)(N+1) \end{array} \end{aligned}$$

32 $$\begin{aligned}&\begin{array}{lll} R^2_{ii}=600 &{} \quad i=1, \ldots , mN, \\ R^2_{ii}=1 &{} \quad i=mN+1, \ldots , 2mN \end{array} \end{aligned}$$

Note that for the above values of $R^1, R^2$, the slides of the specimen show an asymmetric behaviour, namely the displacement is flatter where the material function is bigger (that is, when $R_{ii}(x)=600$).

In Figs. 9 and 10, we report the results when $R^1,R^2$ are the discretization of the following function

33 $$\begin{aligned} \begin{array}{lll} r(x,y)=400exp(y), &{}\quad \text{ for } x\le N \\ r(x,y)=400y &{} \quad \text{ otherwise } \end{array} \end{aligned}$$

Note that in Fig. 10, the boundary datum is chosen as $g_3(t)=\frac{1}{100}cos(2(y-0.5))(x-0.5).$ As expected due to the choice of $R^1, R^2$, we remark an asymmetric behaviour of the fracture in the *y* direction, namely the specimen brakes before where the material function is higher.

### Comparison with GIST

In this section, we present the result of experiments to compare the performance of Algorithm 1 with the following two other algorithms for nonconvex and nonsmooth minimization. We first compare with the GIST “General Iterative Shrinkage and Thresholding” algorithm for $\ell ^\tau$, $\tau <1$ minimization. We took advantage of the fact that for GIST^1^ an open source toolbox is available, which facilitated an unbiased comparison. Moreover, in [25], several tests were made to compare GIST and IRLS “Iteratively reweighted least squares”, showing that the two algorithms have nearly the same performance, with only significant difference in speed, where GIST appears to be the faster one.

Concerning $\ell ^1$-minimization based algorithms, we compared our algorithm with the FISTA “Fast Iterative Shrinkage-Thresholding Algorithm”, see Sect. 3.2.

We remark that the results of [25] show no particular differences in the performance of the algorithm for different values of $\tau$, except that the speed becomes much worse for p near to 1, say $\tau =0.9$. Motivated also by this observations, the comparisons explained in the following were made for one fixed value of $\tau$.

The comparison is carried out through the following three examples, the academical M-matrix problem, an optimal control problem, and a microscopy imaging reconstruction example.

The monotone algorithm is stopped when the $\ell ^\infty$-residue of the optimality condition (9) is of the order of $10^{-3}$ in the *M*-matrix and optimal control problems and of the order of $10^{-8}$ in the imaging example. GIST is terminated if the relative change of the two consecutive objective function values is less than $10^{-5}$ or the number of iterations exceeds 1000. We remark that no significant changes were remarked by setting a lower tolerance than $10^{-5}$ or a bigger number of maximal iteration for GIST.

Since both GIST and the FISTA solve the problem (1) when the operator $\varLambda$ coincides with the identity, we also make this choice in the following subsections. Finally, we remark that the three examples were analysed already in [20] with different aims.

#### M-Matrix Example

We consider

34 $$\begin{aligned} \min _{x \in {\mathbb {R}}^{n\times n}}J(x)= \min _{x \in {\mathbb {R}}^{n\times n}}\frac{1}{2}|A x-b|_2^2+\lambda |x|^\tau _\tau , \end{aligned}$$

*A* is the forward finite difference gradient $A=\left[ G_1', G_2'\right] ',$ with $G_1:=I \otimes D \in {\mathbb {R}}^{n(n+1)\times n^2}$ and $G_2:=D \otimes I \in {\mathbb {R}}^{n(n+1)\times n^2}$, *I* is the $n\times n$ identity matrix, $\otimes$ the tensor product, $D=(n+1)\tilde{D},$ and $\tilde{D}:=diag(ones(n+1,1))+diag(-ones(n,1)-1) \in {\mathbb {R}}^{(n+1)\times n}$, without the last column. Then, $A^T A$ is an *M* matrix coinciding with the 5-point star discretization on a uniform mesh on a square of the Laplacian with Dirichlet boundary conditions. Moreover, (34) can be equivalently expressed as

35 $$\begin{aligned} \min _{x \in {\mathbb {R}}^{n\times n}}\frac{1}{2}|A x|_2^2-(x,f)+\lambda |x|^\tau _\tau , \end{aligned}$$

where $f=A^T b$. If $\lambda =0$, this is the discretized variational form of the elliptic equation

36 $$\begin{aligned} -\varDelta y=f \text{ in } \varOmega , \quad y=0 \text{ on } \partial \varOmega . \end{aligned}$$

For $\lambda >0$, the variational problem (35) gives a sparsity-enhancing solution for the elliptic equation (36), that is, the displacement *y* will be 0 when the forcing *f* is small. Our tests are conducted with *f* chosen as discretization of $f=10 x_1\text{ sin }(5x_2) \text{ cos }(7 x_1)$. The initialization is chosen as the solution of the corresponding non-sparse optimization problem.

We remark that in [37] and [20], the algorithm was also tested in the same situation for different values of $\tau$ and $\lambda$, showing, in particular and consistent with our expectations that the sparsity of the solution increases with $\lambda$.

Here, we focus on the comparison between the performances of Algorithm 1 and GIST. In order to compare the two schemes, we focus on the value of the unregularized functional *J* in (34) reached by both algorithms, the time to acquire it, and the number of iterations. Our tests were conducted for $\tau =0.5$, and $\lambda$ incrementally increasing from $10^{-3}$ to 0.3. The parameter $\varepsilon$ was decreased from $10^{-1}$ to $10^{-6}$. We report the values in Table 1 for $\lambda =0.05$, since for the other values of $\lambda$, the results we obtained are comparable.

We observe that Algorithm 1 achieves always lower values of the functional J, but in a longer time. The number of iterations needed by Algorithm 1 is smaller than the number of iterations of GIST for small values of $\lambda$, more precisely for $\lambda <0.1$. Note that for smaller $\lambda$ the number of iterations of Algorithm 1 is smaller than the one of GIST. This suggests, consistent with our expectation, that the monotone scheme is slower than GIST mainly because it solves a nonlinear equation at each iteration.

We carried out a further test in order to measure the timing performance of Algorithm 1, that is, the algorithm is stopped as soon as the value of J achieved by GIST is reached. In Table 1, we report the time, the number of iterations, the values of J, and the value of $\varepsilon$ reached. We observe that the time is almost always smaller than the one of GIST.

**Table 1:** M-matrix example, $\lambda =0.05$. (a) Comparison between value of functional, time and iterations between Algorithm 1 and GIST. (b) Value of iteration and time to which Algorithm 1 overcome GIST’s value of the functional

(a)			
$\hbox {J}_{{\mathrm{GIST}}}$	264.232	$\hbox {J}_{{\mathrm{mon}}}$	263.92
Time$_{{\mathrm{GIST}}}$	0.701	Time$_{{\mathrm{mon}}}$	26.142
Iterations$_{{\mathrm{GIST}}}$	384	Iterations$_{{\mathrm{mon}}}$	361
(b)			
$\hbox {J}_{{\mathrm{GIST}}}$	264.232	Time$_{{\mathrm{mon}}}$	0.39
Iter$_{{\mathrm{mon}}}$	5	Time$_{{\mathrm{GIST}}}$	0.701

#### Optimal Control Problem

We consider the linear control system

$$\begin{aligned} \frac{\mathrm{d}}{\mathrm{d}t} y(t)=\mathcal {A} y(t)+B u(t), \quad y(0)=0, \end{aligned}$$

that is,

37 $$\begin{aligned} y(T)=\int _0^T e^{\mathcal {A}(T-s)} B u(s) \mathrm{d}s, \end{aligned}$$

where the linear closed operator $\mathcal {A}$ generates a $C_0$-semigroup $e^{\mathcal {A}t}$, $t\ge 0$ on the Hilbert space *X*. More specifically, we consider the one-dimensional controlled heat equation for $y=y(t,x)$:

38 $$\begin{aligned} y_t=y_{xx}+b_1(x)u_1(t)+b_2(x)u_2(t), \quad x \in ]0,1[, \end{aligned}$$

with homogeneous boundary conditions $y(t,0)=y(t,1)=0$ and thus $X=L^2(]0,1[)$. The differential operator $\mathcal {A}y=y_{xx}$ is discretized in space by the second-order finite difference approximation with $n=49$ interior spatial nodes ($\varDelta x=\frac{1}{50}$). We use two time-dependent controls $\overrightarrow{u}=(u_1,u_2)$ with corresponding spatial control distributions $b_i$ chosen as step functions: $b_1(x)=\chi _{].2,.3[}, b_2(x)=\chi _{].6,.7[}.$ The control problem consists in finding the control function $\overrightarrow{u}$ that steers the state $y(0)=0$ to a neighbourhood of the desired state $y_d$ at the terminal time $T=1$. We discretize the problem in time by the mid-point rule, i.e.

39 $$\begin{aligned} A \overrightarrow{u}=\sum _{k=1}^m e^{\mathcal {A}\left( T-t_{k}-\frac{\varDelta t}{2}\right) } (B \overrightarrow{u})_k \varDelta t, \end{aligned}$$

where $\overrightarrow{u}=(u_1^1,\ldots , u_1^m,u_2^1,\ldots u_2^m)$ is a discretized control vector whose coordinates represent the values at the mid-point of the intervals $(t_k,t_{k+1})$. Note that in (39), we denote by *B* a suitable rearrangement of the matrix *B* in (37) with some abuse of notation. A uniform step-size $\varDelta t=\frac{1}{50}$ ($m=50$) is utilized. The solution of the control problem is based on the sparsity formulation (1), where $\varLambda =I$ and $\phi _{\lambda , \tau }(x)=\lambda |x|^{\tau }$ and *b* in (1) is the discretized target function chosen as the Gaussian distribution $y_d(x)=0.4\,\text{ exp }(-70(x-.7)^2))$, centred at $x=.7$. That is, we apply our algorithm for the discretized optimal control problem in time and space where *x* from (1) is the discretized control vector $u \in {\mathbb {R}}^{2m}$, which is mapped by *A* to the discretized output *y* at time 1 by means of (39). Moreover, *b* from (1) is the discretized state $y_d$ with respect to the spatial grid $\varDelta x$. The parameter $\varepsilon$ was initialized with $10^{-3}$ and decreased down to $10^{-8}$.

Similarly as in the previous subsection, we compare the values of the functional, the time and the number of iterations. The experiments are carried out for $\tau =0.5$ and $\lambda$ in the interval $10^{-3}$-0.2. We report only the values for the second control $u_2$ since the first control $u_1$ is always zero (as expected).

As can be seen from Table 2, the same kind of remarks as in the previous subsection apply. In particular, GIST is faster but less precise than Algorithm 1, but Algorithm 1 overcomes the value reached by GIST more rapidly. Note that we reported again only the results we obtained for the two values of $\lambda =0.001$ and $\lambda =0.01$ since for the other values of $\lambda$ tested, we got comparable results.

**Table 2:** Optimal control problem. (a) and (b) Comparison between the value of J, time, iteration of Algorithm 1 and GIST. (c) Value of *J*, iterations, time for which Algorithm 1 overcomes GIST’s

$\lambda$	0.001	0.01
(a)		
$\hbox {J}_{{\mathrm{GIST}}}$	0.073	0.599
Time$_{{\mathrm{GIST}}}$	0.047	0.04
Iterations$_{{\mathrm{GIST}}}$	157	3
(b)		
$\hbox {J}_{{\mathrm{mon}}}$	0.068	0.185
Time$_{{\mathrm{mon}}}$	15.140	14.866
Iterations$_{{\mathrm{mon}}}$	28	32
(c)		
$\hbox {J}_{{\mathrm{mon}}}$	0.071	0.185
Iter$_{{\mathrm{mon}}}$	1	5
Time$_{{\mathrm{mon}}}$	0.1	0.39
Time$_{{\mathrm{GIST}}}$	0.047	0.04

#### Compressed Sensing Approach for Microscopy Image Reconstruction

We compare Algorithm 1 and GIST in a microscopy imaging problem, in particular we focus on the STORM (stochastic optical reconstruction microscopy) method, based on stochastic switching and high-precision detection of single molecules to achieve an image resolution beyond the diffraction limit. The literature on the STORM has been intensively increasing, see e.g. [38–41]. We refer in particular to [20] for a detailed description of the method and for more references.

Our approach is based on the following constrained-minimization problem:

40 $$\begin{aligned} \min _{x \in {\mathbb {R}}^n} |x|^\tau _{\tau } \quad \text{ such } \text{ that } \, \,|A x-b|_2 \le \varepsilon , \end{aligned}$$

where $\tau \in ]0,1]$, *x* is the up-sampled, reconstructed image, *b* is the experimentally observed image, and *A* is the impulse response (of size $m\times n$, where *m* and *n* are the numbers of pixels in *b* and *x*, respectively). *A* is usually called the point spread function (PSF) and describes the response of an imaging system to a point source or point object. Problem (40) can be reformulated as:

41 $$\begin{aligned} \min _{x \in {\mathbb {R}}^n} \frac{1}{2}|Ax-b|^2_2+\lambda |x|^\tau _\tau . \end{aligned}$$

First, we tested the procedure for same resolution images, in particular the conventional and the true images are both $128\times 128$ pixel images. Then, the algorithm was tested in the case of a $16\times 16$ pixel conventional image and a $128 \times 128$ true image. The values for the impulse response *A* and the measured data *b* were chosen according to the literature, in particular *A* was taken as the Gaussian PSF matrix with variance $\sigma =8$ and size $3\times \sigma =24$, and *b* was simulated by convolving the impulse response *A* with a random 0-1 mask over the image adding a white random noise so that the signal to noise ratio is .01.

**Fig. 11:**
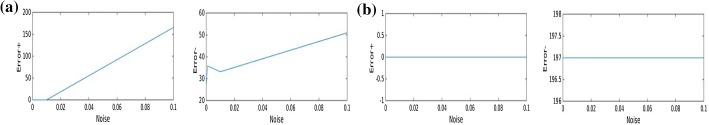
Error$+$ (surplus of emitters), Error− (missed emitters) against noise. **a** Results obtained by Algorithm 1. **b** Results obtained by GIST. $\tau =.5, \lambda =10^{-6}$

**Fig. 12:**
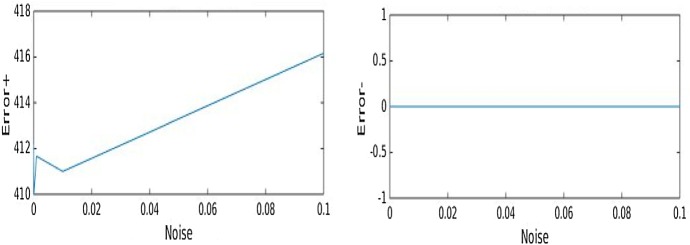
Error$+$ (surplus of emitters), Error− (missed emitters) against noise. Results obtained by the FISTA, $\lambda =10^{-6}$

We carried out several tests with the same data for different values of $\tau ,\lambda$. We report only our results for $\tau =.1$ and $\lambda =10^{-6}, \lambda =10^{-9}$ for the same and the different resolution case, respectively, since for these values the best reconstructions were achieved. We focus on two different types of images, a sparse 0-1 cross-like image and the standard phantom image. In order to compare the performance of Algorithm 1 and the GIST algorithm, we focus on the number of surplus emitters (Error$+$) and missed emitters (Error−) recovered in the case of the cross image and different resolution. The errors are computed on an average over six recoveries for different values of the noise. The graphics of the errors against the noise are reported in Figs. 11 and 12 for Algorithm 1 and GIST, respectively. We remark that these quantities are typically used as a measure of the efficacy of the reconstruction method, see for example [42] (where, under certain conditions, a linear decay with respect to the noise is proven) and [43].

The results shows that by GIST the Error− is always 197, whereas by Algorithm 1 is always under 53 and even smaller for small values of the noise. On the other hand, the Error$+$ by GIST is always 0 and by Algorithm 1 is zero for small values of the noise and then monotonically increasing until it reaches 175 when the noise is equal to 0.1. Consistently with what expected, by Algorithm 1, the graphics show a linear decay w.r.t. the noise, differently from the behaviour showed by GIST. Moreover, the results found by Algorithm 1 lead to more accuracy in the recovery, in the sense that the quantity of missed emitters is smaller, whereas on the other hand, GIST seems to lead to a more sparser solutions (since the Error$+$ is 0 by GIST).

Finally, we remark that in the case of the cross image, GIST is faster than our algorithm, consistently with the result presented in the previous subsection and as expected, since our algorithm solves a nonlinear equation for each minimization problem. On the other hand, in the case of the standard phantom image, GIST results to be far slower than Algorithm 1.

In Fig. 12, we report the results obtained in the same situation by the FISTA “Fast Iterative Shrinkage-Thresholding Algorithm” for $\ell ^1$ minimization. We remark that by the FISTA, the Error$+$ is always above 400, whereas by Algorithm 1 is zero for small value of the noise. This shows that Algorithm 1 leads to more sparsity with respect to the FISTA, consistently with our expectation since the FISTA is based on $\ell ^1$ minimization.

## Perspectives and Open Problems

An open problem of interest to us is the study of problems like (1) for the case where the linear mapping *A* is replaced by a nonlinear, smooth operator $f:{\mathbb {R}}^n \mapsto {\mathbb {R}}^m$. One of the motivations arises from control of nonlinear dynamical system. We could proceed by iteratively applying the monotone scheme in Sect. 2.3 to auxiliary problems arising from linearization of *f* at the current iterate and by updating the sequence such obtained in an outer loop.

We are particularly interested in the case in which the nonlinear operator *f* is nonconvex. From a fracture mechanics point of view, this would mean considering not only small-strain energy as in the current paper, but possibly polyconvex strain energy as in [44]. Considering a nonconvex energy would be more consistent from a mechanical point of view, and in particular in line with Coleman–Noll’s theorem.

## Conclusions

We have developed a monotone convergent algorithm for a class of nonconvex nonsmooth optimization problems arising in the modelling of fracture mechanics and in imaging reconstruction, including the $\ell ^\tau , \tau \in ]0,1]$, the smoothly clipped absolute deviation and the minimax concave penalty. Theoretically, we established the existence of a minimizer of the original problem under assumptions implying coercivity of the functional. Then, we derived necessary optimality conditions for a regularized version of the original problem. The optimality conditions for the regularized problem were solved through a monotonically convergent scheme based on an iterative procedure. We proved the convergence of the iteration procedure under the same assumptions that guarantee existence. A remarkable result is the strict monotonicity of the functional along the sequence of iterates generated by the scheme. Moreover, we proved the convergence of the regularized problem to the original one, as the regularization parameter goes to zero.

The procedure is very efficient and accurate. The efficiency and accuracy of the procedure was verified by numerical tests simulating the evolution of cohesive fractures and microscopy imaging. An issue of high relevance to us was the comparison of the scheme to two alternative algorithms, the GIST “General Iterative Shrinkage and Thresholding” algorithm for $\ell ^\tau$ minimization, with $\tau$ strictly positive and less than 1 and the FISTA “Fast Iterative Shrinkage-Thresholding Algorithm” for $\ell ^1$ minimization. We first compared with GIST by focusing on the infimal value reached by the iteration procedure and on the computing time. Our results showed that the monotone algorithm is able to reach a smaller value of the objective functional when compared to GIST’s, therefore leading to a better accuracy. Finally we compared our scheme with FISTA in sparse recovery related to microscopy imaging. The results showed that the monotone scheme leads to more sparsity with respect to FISTA, as expected since FISTA concerns $\ell ^1$ minimization.

## Footnotes

1 The reference paper is [36], the toolbox can be found in https://github.com/rflamary/nonconvex-optimization.
